# Blind Additive Gaussian White Noise Level Estimation from a Single Image by Employing Chi-Square Distribution †

**DOI:** 10.3390/e24111518

**Published:** 2022-10-24

**Authors:** Zhicheng Wang, Qing An, Zifan Zhu, Hao Fang, Zhenghua Huang

**Affiliations:** 1 School of Electrical and Information Engineering, Wuhan Institute of Technology, Wuhan 430205, China; 2 Artificial Intelligence School, Wuchang University of Technology, Wuhan 430223, China; 3 School of Electronic Information Engineering, Wuhan Donghu University, Wuhan 430212, China

**Keywords:** image patches, additive Gaussian white noise (AGWN) level estimation, Chi-square distribution, AGWN removal

## Abstract

The additive Gaussian white noise (AGWN) level in real-life images is usually unknown, for which the empirical setting will make the denoising methods over-smooth fine structures or remove noise incompletely. The previous noise level estimation methods are easily lost in accurately estimating them from images with complicated structures. To cope with this issue, we propose a novel noise level estimation scheme based on Chi-square distribution, including the following key points: First, a degraded image is divided into many image patches through a sliding window. Then, flat patches are selected by using a patch selection strategy on the gradient maps of those image patches. Next, the initial noise level is calculated by employing Chi-square distribution on the selected flat patches. Finally, the stable noise level is optimized by an iterative strategy. Quantitative, with association, to qualitative results of experiments on synthetic real-life images validate that the proposed noise level estimation method is effective and even superior to the state-of-the-art methods. Extensive experiments on noise removal using BM3D further illustrate that the proposed noise level estimation method is more beneficial for achieving favorable denoising performance with detail preservation.

## 1. Introduction

In image denoising, additive Gaussian white noise level is an important parameter, but it is usually unknown in real-life images [1,2]. A denoising method with accurate noise levels may generate comfortable results with abundant richness [3]. The prior ways usually set it empirically, which may result in undesirable maps: the method with a high noise level may over-smooth rich structures, while AGWN may still exist in the denoising result with a low level [4,5].

To provide accurate noise levels, researchers have developed a number of noise level estimation methods [6,7]. Among them, the patch-based methods are widely used due to their easy implantation and low computational burden. However, noise levels may be overestimated or underestimated with the usage of inaccurate homogeneous patches, which are sensitive to the complexity of images and noise levels.

**Figure 1:**
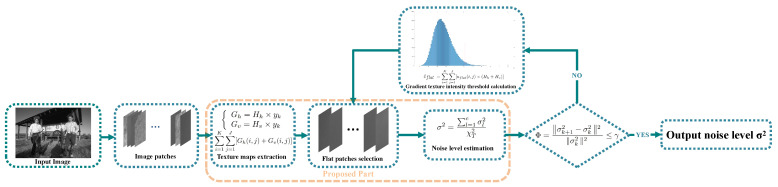
Flowchart of the proposed noise level estimation method.

To cope with the problems, this paper presents a novel method of noise level estimation, which combines a simple and effective patch-based method with the Chi-square distribution. Figure 1 shows the flowchart of our proposed method. The input image is first divided into image patches through a sliding window. Then, we select the flat patches using a flat patch selection strategy with the texture maps extraction. Next, the initial noise level is calculated by employing Chi-square distribution on the selected flat patches. Finally, the estimation performance is boosted via an iterative strategy. The main contributions are detailed as follows:

1. A novel patch-based noise level estimation method based on Chi-square distribution is proposed.
2. An optimization iteration scheme is proposed to improve the accuracy and stability of the noise level estimation strategy.
3. Quantitative results associated with the qualitative results of the experiments are used to verify the effectiveness of the proposed noise level strategy.

The remainder of this paper is organized as follows. Section 2 elaborates the literature of existing noise level estimation methods. The proposed noise level estimation method is described in Section 3. Section 4 shows our experimental results, and we conclude this paper in Section 5.

## 2. Literature Review

Currently, the noise level estimation methods are roughly divided into the following three categories:

- **Filter-based methods:** These methods extract the differential image by convolving the noisy image with a specially designed filter, and then use the filtered differential image as the noise map to estimate the noise level [8,9]. For example, Immerkaer [10] designed an image structure insensitive method to filter noisy images and estimate noise level by averaging the convolved images. It performed well on estimating noise levels, but lost in structural images [11]. To address this issue, Rank et al. [12] combined histogram statistics with a filter-based approach to generate the stable noise level. However, it produced an overestimation noise level from texture images [13]. To reduce the adverse effects caused by the image structures, Tai et al. [14,15] applied a Laplacian operator to remove strong edge pixels before filtering so as to improve the accuracy at low noise levels.
- **Transform-based methods:** Instead of using spatial information, these methods estimate noise level by transforming an image into other spaces [16,17]. For example, Donoho [18] proposed a mean absolute deviation (MAD) method to estimate the noise level on the wavelet domain. They treated all the coefficients of the highest frequency subband as noise, and estimated the standard deviation. This method performed well on estimating high noise level, but the error is increased when noise level is low [19,20]. Recently, the models based on the singular value decomposition (SVD) were widely used in noise level estimation [21,22]. For example, Wei et al. [23] used singular value tail data in SVD to estimate the noise level, which minimizes the interference of image structures. However, image details and noise cannot be completely separated at the end of the singular value for images with rich structures, so the noise level is invariably overestimated [24].
- **Patch-based methods:** In these methods, a noisy image is initially decomposed into a group of patches. Then, the homogeneous patches are selected via various statistical techniques for noise level estimation [25,26,27]. For example, Pyatykh et al. [28] proposed a method based on principal component analysis (PCA), which viewed the smallest eigenvalue of the image patch covariance matrix as the noise level. Since the minimum eigenvalue of PCA about the noisy image patch does not always satisfy the null hypothesis, it is easy to cause instability or overestimation of the noise level estimation [29,30,31]. In order to optimize the above methods, Liu et al. [32] proposed an automatic noise level estimation method by adaptively selecting effective image patches for covariance calculation. It effectively reduces the obvious overestimation of the low noise level based on the PCA method, but there is still underestimation in the case of high noise level [33].

The above mentioned methods have achieved considerable progress in improving the accuracy of noise level estimation, but they also have their respective drawbacks [34,35]. For example, filter-based methods have a large error when the structures in an image are dense and have a highly computational complexity [36,37]. Transform-based methods always produce over-estimation results when noise levels are low [38,39]. Patch-based methods may generate underestimation results at high noise levels [17,40,41].

## 3. Image Noise Level Estimation Based on Chi-Square Distribution

### 3.1. Image Decomposition into Patches

Assuming that an image is degraded by additive white Gaussian noise (AWGN) with unknown standard deviation σ, the model can be defined as

(1) Y=X+N,

where *X* is the latent clean image, *N* is AWGN: $N∼N(0,\sigma^{2})$, and *Y* is the noisy image. For a pure flat image, the contaminated image $Y_{F}$ can be expressed as

(2) $$Y_{F}=X_{F}+N,$$

where $X_{F}$ represents the ground truth image. As $X_{F}$ is purely flat, its gradient maps are zero. Then the noise level of the noisy image $Y_{F}$ can be easily obtained by the following formula:

(3) $$\sigma_{f}=\sqrt{\frac{\sum_{i=1}^{P}\sum_{j=1}^{Q}{\left(y\left(i,j\right)-{\bar{Y}}_{F}\right)}^{2}}{Num}},$$

where *P* and *Q* are the height and width of the noisy image $Y_{F}$, y(i,j) represents gray value of $Y_{F}$ at the point (i,j), ${\bar{Y}}_{F}$ is the average of $Y_{F}$, Num is the number of pixels, and $\sigma_{f}$ is the estimated noise level. Unfortunately, natural images usually have rich structures, so it is inaccurate to estimate the noise level directly using Equation (3). To accurately estimate the noise level of a degraded image, we first decompose the image into many image patches through a sliding window (such as 6×6 in this paper). The patch $y_{k}$ can be defined as

(4) $$y_{k}=x_{k}+n_{k},\;\;\;\;\;\;\;k=1,2,3,\cdots ,S,$$

where *S* is the number of image patches. $x_{k}$ is the *k*-th clear patch from *X*, and each patch is defined by its center pixel. $y_{k}$ denotes noisy patch corrupted by Gaussian noise $n_{k}$ with zero-mean and noise level $\sigma_{n}$. For the follow-up work, we used the set $\left\{Y_{k}\right\}{|}_{k=1}^{S}$ to represent the noisy patches, which can be subdivided into contaminated detail and latent flat patches.

### 3.2. Flat Patches Selection

In this work, we will select latent flat patches from set $\left\{Y_{k}\right\}{|}_{k=1}^{S}$ and define them as set $\left\{Z_{l}\right\}{|}_{l=1}^{C}$, where *C* represents the number of flat patches. Comparing the details and flat of the patches, we find that the flatness of the patches can be well expressed by the gradient features maps.

The gradient maps of $y_{k}$ can be calculated with the following function

(5) $$\left\{\begin{array}{c} G_{h}=H_{h}\times y_{k}\\ G_{v}=H_{v}\times y_{k} \end{array},\right.$$

where $H_{h}$ and $H_{v}$ are the horizontal *h* and vertical *v* gradient matrices, respectively. $G_{h}$ and $G_{v}$ respectively represent the horizontal and vertical gradient strength maps. By substituting Equation (4) into Equation (5), it is rewritten as

(6) $$\left\{\begin{array}{c} G_{h}=H_{h}\times \left(x_{k}+n_{k}\right)\\ G_{v}=H_{v}\times \left(x_{k}+n_{k}\right) \end{array}.\right.$$

The gradient texture intensity $\varepsilon_{k}$ of the image patch $y_{k}$ is formulated as

(7) $$\begin{array}{cc} \varepsilon_{k}= & \sum_{i=1}^{K}\sum_{j=1}^{J}\left[G_{h}\left(i,j\right)+G_{v}\left(i,j\right)\right]\\ = & \sum_{i=1}^{K}\sum_{j=1}^{J}\left[H_{h}\times \left(x_{k}\left(i,j\right)+n_{k}\left(i,j\right)\right)+H_{v}\times \left(x_{k}\left(i,j\right)+n_{k}\left(i,j\right)\right)\right]\\ = & \sum_{i=1}^{K}\sum_{j=1}^{J}\left[\left(H_{h}+H_{v}\right)\times x_{k}\left(i,j\right)+\left(H_{h}+H_{v}\right)\times n_{k}\left(i,j\right)\right], \end{array}$$

where *K* and *J* respectively represent the height and width of the image patch $y_{k}$. In order to set a reasonable threshold to select flat patches, we conduct a more detailed study on the gradient texture intensity $\varepsilon_{k}$. To simplify the process, Equation (7) can be rewritten as

(8) $$\varepsilon_{k}=A\left(x_{k}\right)+B\left(n_{k}\right),$$

where $x_{k}$ is the latent clean patch, $A\left(x_{k}\right)=\sum_{i=1}^{K}\sum_{j=1}^{J}\left[\left(H_{h}+H_{v}\right)\times x_{k}\left(i,j\right)\right]$, $n_{k}$ represents the Gaussian noise of patch and $B\left(n_{k}\right)=\sum_{i=1}^{K}\sum_{j=1}^{J}\left[\left(H_{h}+H_{v}\right)\times n_{k}\left(i,j\right)\right]$. The gradient texture intensity component $A(x_{k})$ is determined by the patch $x_{k}$ and $B(n_{k})$ is generated by AGWN. For flat patches from $\left\{Z_{l}\right\}{|}_{l=1}^{C}$, $x_{l}$ is absolutely flat so that A(x)=0. Therefore, the gradient texture intensity $\varepsilon_{flat}$ of the patch can be expressed as

(9) $$\begin{array}{cc} \varepsilon_{flat} & =A\left(x_{flat}\right)+B\left(n_{flat}\right)=B\left(n_{flat}\right)\\ & =\sum_{i=1}^{K}\sum_{j=1}^{J}\left[n_{flat}\left(i,j\right)\times \left(H_{h}+H_{v}\right)\right], \end{array}$$

where $x_{flat}$ is the latent flat patch and $n_{flat}$ is AGWN. Since $B(n_{k})$ is only affected by AGWN and patch size, we can fit on the extremely flat image with known conditions to calculate the gradient texture intensity threshold. Firstly, the image is divided into patches, and then the gradient texture intensity of each patch is counted by Equation (9). The gradient texture intensity threshold $\varepsilon_{\delta }$ can be calculated from

(10) $$\varepsilon_{\delta }=Max\left(\left\{B(n_{k}\left(i,j\right))\right\}\right),$$

where $B(n_{k}\left(i,j\right))$ represents the noise gradient texture intensity component of the noise patch $n_{k}\left(i,j\right)$. According to the statistics of a large number of noise patches, the maximum value of $B(n_{k}\left(i,j\right))$ is selected as $\varepsilon_{\delta }$. So we have

(11) $$\varepsilon_{flat}<\varepsilon_{\delta }<\varepsilon .$$

Equation (11) shows that the left side is a good approximation of the gradient texture intensity of the flat patches. In the selection of the flat patch on the natural image, we will use the parameter $\varepsilon_{\delta }$ as the threshold. If the gradient texture intensity $\varepsilon_{k}$ is less than the threshold $\varepsilon_{\delta }$, such that the patch can be regarded as a flat patch and used for noise level estimation. For the patch $z_{l}$ in the selected flat patch set $\left\{Z_{l}\right\}{|}_{l=1}^{C}$, we have

(12) $$z_{l}=x_{l}+n_{l},$$

where $z_{l}$ is the latent flat patch of the noisy image *Y*, $x_{l}$ is the flat patch of *X*, and $n_{l}$ is AGWN.

### 3.3. Image Noise Level Estimation

Ideally, according to Equation (12), since $z_{l}$ is the flat image patch, its average is equal to the mean of the clean patch $x_{l}$, and the variance is provided by the noise $n_{l}$. We can draw the conclusions

(13) $$Z∼N\left(\mu ,\sigma^{2}\right)=\left\{\begin{array}{c} \;z_{1}∼N\left(\mu_{1},\sigma_{1}^{2}\right)\\ \;z_{2}∼N\left(\mu_{2},\sigma_{2}^{2}\right)\\ \;\;\vdots \\ \;z_{C-1}∼N\left(\mu_{C-1},\sigma_{C-1}^{2}\right)\\ \;z_{C}∼N\left(\mu_{C},\sigma_{C}^{2}\right) \end{array},\right.$$

where μ is the average of all patches $\left\{Z_{l}\right\}{|}_{l=1}^{C}$, $\mu_{l}$(l∈(1,C)) is the mean of the *l*-th flat patch $z_{l}$, and $\sigma_{l}^{2}$ is the variance of the *l*-th flat patch $z_{l}$. Equation (13) can be deduced as

(14) $$\left(Z-\mu \right)∼N\left(0,\sigma^{2}\right)=\left\{\begin{array}{c} \;(z_{1}-\mu_{1})∼N\left(0,\sigma_{1}^{2}\right)\\ \;(z_{2}-\mu_{2})∼N\left(0,\sigma_{2}^{2}\right)\\ \;\;\vdots \\ \;(z_{C-1}-\mu_{C-1})∼N\left(0,\sigma_{C-1}^{2}\right)\\ \;(z_{C}-\mu_{C})∼N\left(0,\sigma_{C}^{2}\right) \end{array}.\right.$$

In the ideal case, $\sigma^{2}=\sigma_{1}^{2}=\sigma_{2}^{2}=\cdots =\sigma_{C}^{2}$, so Equation (14) is simplified as

(15) $$\left\{Z_{l}-\mu_{l}\right\}\left|{}_{l=1}^{C}∼N\left(0,\sigma^{2}\right)\right..$$

Assuming $M_{l}=\left(Z_{l}-\mu_{l}\right)$, $M_{1}$, $M_{2}$, ⋯, $M_{C}$ are mutually independent and identically distributed random variables. Then we can obtain

(16) $$G_{l}=\frac{M_{l}}{\sigma^{2}}∼N\left(0,1\right).$$

For all flat patches, the distribution $U=\sum_{l=1}^{C}G_{l}^{2}$ follows the Chi-square distribution with the degree of freedom *C*, and can be denoted as $\chi^{2}\left(C\right)$. Then, the calculation formula of the noise level is defined as follows:

(17) $$\sigma =\sqrt{\frac{\sum_{l=1}^{C}\sigma_{l}^{2}}{\chi_{T}^{2}}},$$

with the confidence level *T*.

### 3.4. Noise Level Estimation Optimization

In order to improve the accuracy of noise level estimation, we use the iterative noise level $\sigma_{t}$ as a parameter in combination with the gradient texture intensity threshold $\varepsilon_{\delta }$ to further select the flat patches $\left\{Z_{l}\right\}{|}_{l=1}^{C}$. As shown in Algorithm 1, first, the initial noise level $\sigma_{0}$ is estimated by the method in Section 3.3. Next, the threshold value $\varepsilon_{\delta }$ is obtained through the gradient feature and iterative noise level $\sigma_{t}$. After, the flat patches $\left\{Z_{l}\right\}{|}_{l=1}^{C}$ are selected according to $\varepsilon_{\delta }$. Then, the noise level $\sigma_{\left(t+1\right)}$ is estimated by Chi-square distribution on the flat patches $\left\{Z_{l}\right\}{|}_{l=1}^{C}$. This process is iterated until the estimated noise level $\sigma_{\left(t+1\right)}$ converges to a fixed point. The iterative convergence criterion is

(18) $$\Phi =\frac{∥\sigma_{t+1}^{2}-\sigma_{t}^{2}∥{}^{2}}{∥\sigma_{t}^{2}∥{}^{2}}\leq \gamma ,$$

where $\gamma =10^{-3}$. $\sigma_{t}$ is the estimation of the noise level in the *t*-th iteration while $\sigma_{t+1}$ is the estimation result in the (*t* + 1)-th iteration. If the termination condition is satisfied, the estimated final noise level $\sigma_{end}$ is output.
**Algorithm 1** Noise level estimation optimization.**Initial input**: Noised Image $Y\subseteq R^{P\times Q}$, Patch Size *d*, Iteration Number Iter, Convergence Threshold γ. **Step 1:** Generating image patch dataset $\left\{Y_{k}\right\}{|}_{k=1}^{S}$, which contains S=(P−c(d+2))×(Q−(d+2)) patches with patch size $w=d^{2}$ from the noisy image *Y*; **Step 2:** Generating the gradient texture intensity dataset $\left\{E_{k}\right\}{|}_{k=1}^{S}$, where $\varepsilon_{k}$ is calculated using Equation (7) from the patch dataset $\left\{Y_{k}\right\}{|}_{k=1}^{S}$; **Step 3**: Estimated initial noise level $\sigma_{0}$ by Section 3.3; **Step 4:** Calculating the threshold $\varepsilon_{\delta }$ combine with $\sigma_{t}$ using Equation (10); // The selection of flat patches // **for** k=1  to  *S* **do**  **if** $\varepsilon_{k}<=\varepsilon_{\delta }$ **then**   The patch $y_{k}$ is regarded as the flat image patch $Z_{l}$;  **else**   The patch $y_{k}$ is considered a detail patch and is removed;  **end if** **end for** // Image noise level calculation // **for** t=0 to Iter **do**  Calculating $\sigma_{\left(t+1\right)}$ with $\left\{Z_{l}\right\}{|}_{l=1}^{C}$ using Equation (17);  Generating Φ using Equation (18);  **if** Φ≥γ **then**   $\sigma_{t}=\sigma_{\left(t+1\right)}$ and back to **step 4**;  **else**   $\sigma_{end}=\sigma_{\left(t+1\right)}$ and **break**;  **end if** **end for****Output**: Estimated noise level $\sigma_{end}$.

## 4. Experimental Results and Discussions

In this section, extensive experiments are conducted to evaluate the performance of our proposed method. In Section 4.1, we first perform simulation experiments by superimposing different noise levels on three synthetic flat images to verify the correctness of our theory. The selection of flat patches and the stability of the iterative strategy are then examined as described in Section 4.2 and Section 4.3. Finally, we compare our model with the state of the art to demonstrate its powerful performance. Furthermore, in Section 4.5 and Section 4.6, we test the practical applicability of our method by combining it with the BM3D denoising method.

The complete experiments of the image noise level estimation algorithms were performed under the MATLAB 2019b software on a Windows 10 with Intel i7 eight-core, CPU 3.0 GHz, and 8 GB RAM memory. In addition, the comparison models used in our experiments, such as Olsen [8], Tai [14], Donoho [18], are generic versions without tuning parameters.

### 4.1. Feasibility Study on Test Flat Images

**Figure 2:**
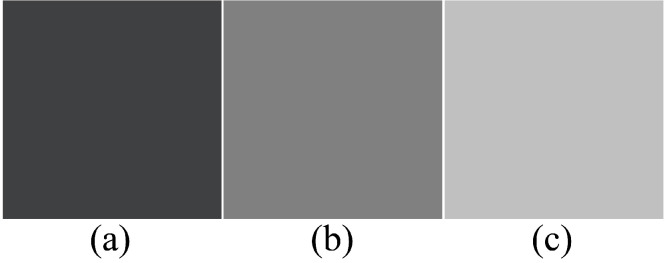
Test flat images with the gray values of (**a**) 64, (**b**) 128, and (**c**) 192, respectively.

To verify the feasibility of the proposed noise level estimation theory, three different flat images shown in Figure 2 are selected to be experimented. These images are 1024×1024 pixels, and the gray values are respectively 64, 128, and 192. The sliding window size for the image patches is 6×6 and $T=1-e^{-6}$.

**Figure 3:**
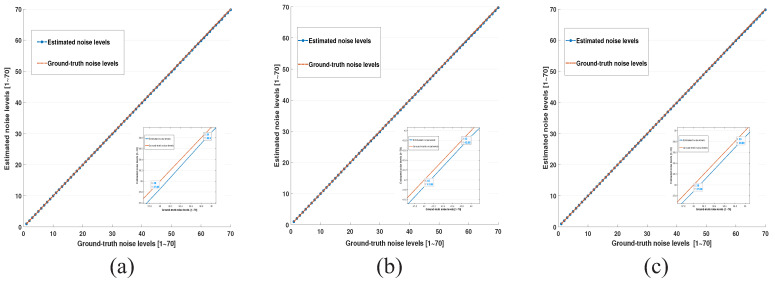
Noise level estimation experiments on test flat images (**a**) 64, (**b**) 128, and (**c**) 192 with different noise levels.

The noise level estimation curves are shown in Figure 3, from which we can see that the noise levels estimated by the proposed scheme are very close to the ground-truth noise levels, indicating that the variance distribution of flat image patches obeys the Chi-square distribution, which validates the feasibility of the proposed method.

### 4.2. Analysis of the Flat Patch Selection

**Figure 4:**
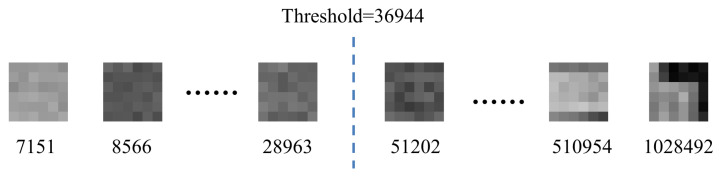
Example patches and the threshold on the Stable image with σ=5.

The accuracy of the flat patches selection is key to our method. Some patches with its gradient texture strength maps and the threshold of noisy image Stable (σ=5) are shown in Figure 4. According to Equation (11), the patches are viewed as flat patches when $\varepsilon_{k}<\varepsilon_{\delta }$ = 36,944, where $\varepsilon_{\delta }$ is calculated by the extremely flat image with iterative noise level $\sigma_{t+1}$.

**Figure 5:**
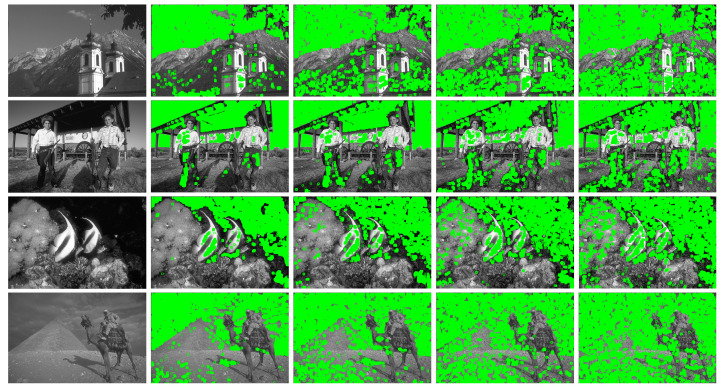
Flat patches selection results of four images (Church, Stable, MoorishIdol and Desert). From left to right: clean images, partition results when σ=5, σ=10, σ=15 and σ=20 for AGWN, where the green region represents the selection results of the flat patches.

As shown in Figure 5, the visual results of four representative images are displayed. We experimented on these images at four different standard noise levels (σ=5,10,15, and 20). One can observe that the image is divided into numerous patches, and the flat patches are shown in the green area of the labeled images. At different noise levels, our method can adaptively select the flat patches, which can be helpful to estimate the noise level more accurately. The method of flat patch selection will be used in Section 4.4, Section 4.5 and Section 4.6.

### 4.3. Discussion of the Iterative Model

In this section, we test the impact of the iterative model on our method. Firstly, the gradient texture intensity threshold $\varepsilon_{\delta }$ is calculated through the iterative noise level $\sigma_{t}$. Secondly, the iterative noise level $\sigma_{t+1}$ is obtained according to the Chi-square distribution. Finally, the final estimated noise level $\sigma_{end}$ is output according to the termination condition Φ and Iter, where Φ is calculated by Equation (18) and Iter=10.

**Figure 6:**
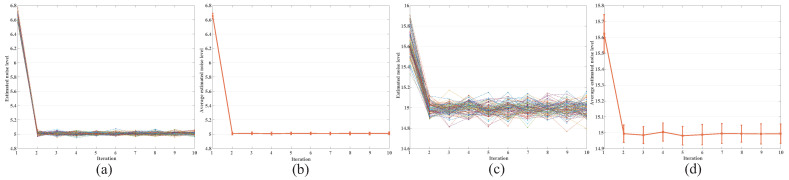
Estimated noise level in each iterative. We conducted 100 experiments. (**a**) Stable image added noise level σ=5. (**b**) Average estimated noise level of Stable, σ=5. (**c**) Stable image added noise level σ=15. (**d**) Average estimated noise level of Stable, σ=15.

As illustrated in Figure 6, they are the experimental results of our optimization method. Figure 6a–c shows the estimated noise level along with iteration on the Stable image respectively with σ=5 and σ=15; both of them were experimented 100 times. Accordingly, Figure 6b–d shows their average estimated noise level along with iteration. These experimental results show that the noise level with the iterative strategy in our method can be converged to a fixed point, which verifies its effectiveness.

### 4.4. Comparisons of Experiments on Synthetic Images

**Figure 7:**
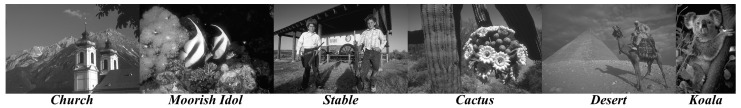
Six well-known testing images in the BSDS database.

We tested the proposed noise level estimation method on six well-known images (which are from the BSD image database [42]) shown in Figure 7. The proposed method is compared with state-of-the-arts, such as Donoho [18], Immerkar [10], Olsen [8], Pyatykh [28] and Tai [14].

**Figure 8:**
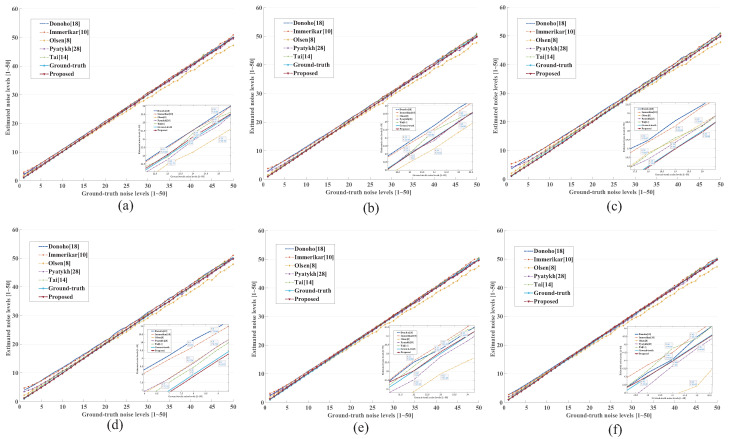
Noise level estimation experiments on synthetic images (**a**) Church, (**b**) MoorishIdol, (**c**) Stable, (**d**) Cactus, (**e**) Desert, and (**f**) Koala with different noise levels.

The comparisons of noise level estimation results are shown in Figure 8, where the noise level range from 1 to 50. We can observe that the proposed method performs better than the state of the art on noise level estimation for each image under the same noise level. In particular, the proposed method can also yield pleasing results on images with rich textures (such as the Koala image shown in Figure 8f. In addition, the Table 1 shows the average error of estimated noise levels from six images, which is calculated as $E_{err}=\left|E_{n}-{\bar{E}}_{n}\right|$. At each noise level, the first two best results are bold. The results show that our method produces smaller average errors in most cases than other advanced noise level estimation methods, demonstrating that our method has powerful capacity in accurately estimating noise level. Compared with the Tai algorithm, our method is slightly inferior when the noise level is 15, where the reason may be that some noised structural patches are mis-viewed as flat patches for noise level estimation. However, our method gains superiority when the noise level is other.

### 4.5. Combined with BM3D on Synthetic Images

**Figure 9:**
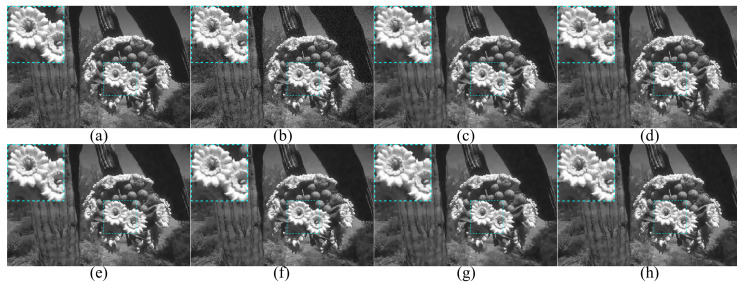
Visual results (**a**) Clean image Cactus; (**b**) Noisy image with σ=10; (**c**–**h**) Visual results of BM3D with noise level estimation in (**c**) [8], (**d**) [10], (**e**) [14], (**f**) [18], (**g**) [28], and (**h**) ours.

**Figure 10:**
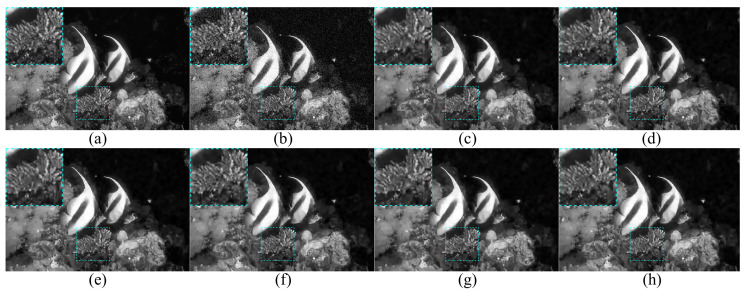
Visual results. (**a**) Clean image MoorishIdol; (**b**) Noisy image with σ=15; (**c**–**h**) Visual results of BM3D with noise level estimation in (**c**) [8], (**d**) [10], (**e**) [14], (**f**) [18], (**g**) [28], and (**h**) ours.

We also test noise level estimations with the classic block-matching and 3D collaborative filtering (BM3D) [43] method for blind AWGN noise removal. Two synthetic images with different noise levels are tested, and the visual results are compared in Figure 9 and Figure 10. However, their differences at each level on each image are small, resulting in the subtle differences of visual results, especially in the enlarged areas. The quantitative denoising results (the peak signal-to-noise ratio (PSNR) [44] is adopted to assess their ability for noise reduction and the structural similarity index (SSIM) [45] is exploited to assess their capability for structure preservation) are respectively presented in Table 2 and Table 3. We combine BM3D with true noise level to obtain the reconstructed image and use its PSNR and SSIM values as benchmarks to evaluate noise level estimation methods. It can be seen that our method tends to produces competitive results that are closer to the benchmarks than the state-of-the-art methods. Generally, combining BM3D with our method produces the best results compared to the images produced by other methods, but the performance on some images (such as Church and Koala) is a little worse than other methods. The reason is that the BM3D method over-smooths the irregular structures because the self-similarity property of these two noised images is not exhibited.

### 4.6. Combined with BM3D on Real-World Images

**Figure 11:**
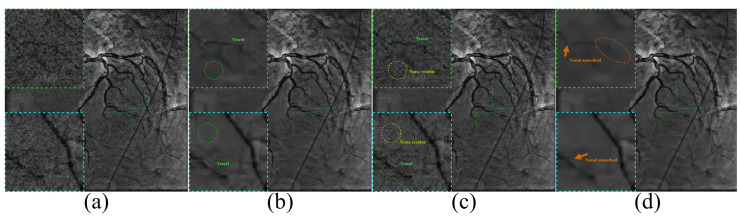
Visual comparison results (**a**) real-life X-ray angiogram at the 57th frame. (**b**) Visual result of BM3D with our noise level estimation method (σ=13.0365). (**c**,**d**) Visual results of BM3D respectively with empirical noise levels 8 and 18.

**Figure 12:**
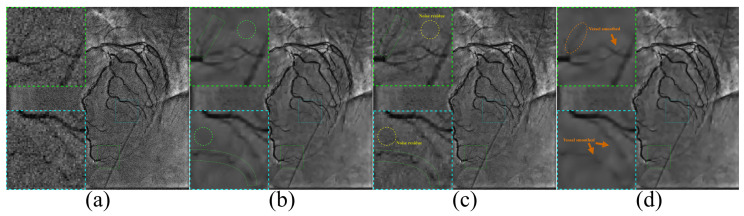
Visual comparison results (**a**) real-life X-ray angiogram at the 85th frame. (**b**) Visual result of BM3D with our noise level estimation method (σ=18.6854). (**c**,**d**) Visual results of BM3D respectively with empirical noise levels 14 and 24.

To further verify the effectiveness of our method, we conduct experiments on real-life X-ray angiograms, as shown in Figure 11 and Figure 12. From the figures, several observations can be concluded: First, when the noise level is empirically set too small, although the blood vessels are preserved well, the noise will be removed incompletely, as shown in Figure 11c and Figure 12c. Second, when the noise level is empirically set too large, the noise is thoroughly removed but the fine vessels are over-smoothed, as shown in Figure 11d and Figure 12d. In contrast, the proposed method can perfectly reduce noise as well as preserving rich vessels, as shown in Figure 11b and Figure 12b. The extensively experimental results illustrate that our method can yield satisfactory results, which are beneficial for applications.

## 5. Conclusions

Image noise levels are unknown in the real-life world, thus how to robustly and accurately estimate the blind noise level from real-world images is important for image denoising methods to remove noise. This paper proposed a novel noise level estimation method by using Chi-square distribution on image patches. The key procedures of the proposed method, including image decomposition into patches, flat patches selection, image noise level estimation based on Chi-square distribution, and robust noise level estimation by an iterative strategy, are discussed in detail. The results of the experiments on flat images, synthetic maps, and real-world images respectively validated the feasibility, the effectiveness, and the further application of the proposed method. Although the proposed method obtained feasible and competitive results, its performance may decrease when the images have rich structures. In the future work, we will further optimize the selection of flat patches to estimate noise levels more accurately.

## Figures and Tables

**Table 1:** The average error value of the images in Figure 7. The bold font indicates the first two best results at each noise level.

Noise Level	5	10	15	20	25	30	35	40	45	50
Donoho [18]	0.7058	0.6157	0.5241	0.5013	0.3696	0.4358	0.3562	0.3248	0.3010	0.3885
J. Immerkar [10]	0.8334	0.6019	0.4510	0.3513	0.2887	0.2427	0.2479	0.2362	**0.2554**	**0.2411**
S. I. Olsen [8]	0.6182	0.5561	0.4901	0.5190	0.4459	0.4680	0.4862	0.4522	0.3838	0.4088
S. Pyatykh [28]	0.5190	0.3329	0.2391	**0.1873**	**0.2160**	0.2960	0.3165	**0.1717**	0.2614	0.2485
Tai Yang [14]	**0.1361**	**0.1999**	**0.1732**	0.2028	0.2247	**0.1696**	**0.2226**	0.2053	0.3556	0.4126
Our proposed	**0.1057**	**0.1802**	**0.1994**	**0.1406**	**0.1312**	**0.1272**	**0.1163**	**0.1326**	**0.0845**	**0.1624**

**Table 2:** Average PSNR scores and its standard deviation of the images in Figure 7; each was experimented 100 times. The bold font denotes the best results.

BM3D [43] +		*Church*			*MoorishIdol*			*Stable*	
Predictive Model	10	20	40	10	20	40	10	20	40
True noise level (benchmark)	34.5386 ± 0.0215	31.0408 ± 0.0364	27.8572 ± 0.0451	32.9813 ± 0.0207	29.4181 ± 0.0325	26.4669 ± 0.0342	32.0914 ± 0.0179	28.2578 ± 0.026 2	25.2087 ± 0.0264
Tai Yang [14]	34.5259 ± 0.0236	31.0271 ± 0.0367	27.8536 ± 0.0456	32.9708 ± 0.0192	29.4011 ± 0.0338	26.4630 ± 0.0350	32.0358 ± 0.0231	28.1978 ± 0.0313	25.1916 ± 0.0292
Donoho [18]	34.4205 ± 0.0254	30.9860 ± 0.0360	27.8447 ± 0.0461	32.7986 ± 0.0220	29.3387 ± 0.0342	26.4538 ± 0.0330	31.6144 ± 0.0296	28.0578 ± 0.0303	25.1678 ± 0.0287
Immeakar [10]	34.4438 ± 0.0232	30.9898 ± 0.0369	27.8449 ± 0.0455	32.8660 ± 0.0177	29.3583 ± 0.0333	26.4505 ± 0.0337	31.6606 ± 0.0251	28.0919 ± 0.0273	25.1670 ± 0.0274
S. I. Olsen [8]	34.5307 ± 0.0216	31.0705 ± 0.0365	27.8540 ± 0.0437	32.9744 ± 0.0207	29.4382 ± 0.0330	26.4795 ± 0.0355	31.9148 ± 0.0320	28.2026 ± 0.0337	25.2394 ± 0.0262
S. Pyatykh [28]	34.5170 ± 0.0232	**31.0326** ± **0.0390**	**27.8602** ± **0.0452**	32.9632 ± 0.0212	29.4093 ± 0.0328	26.4700 ± 0.0337	31.9727 ± 0.0265	28.2078 ± 0.0288	25.2043 ± 0.0282
Our proposed	**34.5359** ± **0.0237**	31.0322 ± 0.0312	27.8526 ± 0.0459	**32.9826** ± **0.0264**	**29.4206** ± **0.0264**	**26.4661** ± **0.0302**	**32.0962** ± **0.0214**	**28.2592** ± **0.0230**	**25.1962** ± **0.0259**
**BM3D [43] +**		***Cactus***			***Desert***			***Koala***	
**Predictive Model**	**10**	**20**	**40**	**10**	**20**	**40**	**10**	**20**	**40**
True noise level (benchmark)	32.1121 ± 0.0208	28.4538 ± 0.0263	25.6292 ± 0.0291	32.9813 ± 0.0207	29.4181 ± 0.0325	26.4669 ± 0.0342	33.9070 ± 0.0185	30.4351 ± 0.0290	27.5316 ± 0.0370
Tai Yang [14]	32.0414 ± 0.0241	28.4121 ± 0.0349	25.6221 ± 0.0289	32.9708 ± 0.0192	29.4011 ± 0.0338	26.4630 ± 0.0350	33.9049 ± 0.0180	30.4268 ± 0.0284	27.5322 ± 0.0366
Donoho [18]	31.6267 ± 0.0259	28.3106 ± 0.0276	25.6093 ± 0.0298	32.7986 ± 0.0220	29.3387 ± 0.0342	26.4538 ± 0.0330	33.8378 ± 0.0219	30.4063 ± 0.0284	27.5333 ± 0.0370
Immeakar [10]	31.7469 ± 0.0227	28.3446 ± 0.0278	25.6079 ± 0.0289	32.8660±0.0177	29.3583 ± 0.0333	26.4505 ± 0.0337	33.8755 ± 0.0193	30.4110 ± 0.0286	**27.5321** ± **0.0369**
S. I. Olsen [8]	32.0035 ± 0.0237	**28.4579** ± **0.0270**	25.6467 ± 0.0296	32.9744±0.0207	29.4382 ± 0.0330	26.4795 ± 0.0355	33.9043 ± 0.0197	30.4461 ± 0.0294	27.4901 ± 0.0372
S. Pyatykh [28]	32.0124 ± 0.0284	28.4252 ± 0.0314	**25.6301** ± **0.0289**	32.9632 ± 0.0212	29.4093 ± 0.0328	26.4700 ± 0.0337	33.9063 ± 0.0183	**30.4358** ± **0.0288**	27.5290 ± 0.0370
Our proposed	**32.1210** ± **0.0190**	28.4463 ± 0.0228	25.6193 ± 0.0342	**32.9826** ± **0.0264**	**29.4206** ± **0.0264**	**26.4661** ± **0.0302**	**33.9071** ± **0.0228**	30.4244 ± 0.0316	27.5426 ± 0.0341

**Table 3:** Average SSIM scores and its standard deviation of the images in Figure 7; each was experimented 100 times. The bold font denotes the best results.

BM3D [43] +		*Church*			*MoorishIdol*			*Stable*	
Predictive Model	10	20	40	10	20	40	10	20	40
True noise level (benchmark)	0.9162 ± 0.0004	0.8542 ± 0.0012	0.7581 ± 0.0019	0.9125 ± 0.0005	0.8251 ± 0.0015	0.6983 ± 0.0032	0.9188 ± 0.0005	0.8175 ± 0.0013	0.6862 ± 0.0020
Tai Yang [14]	0.9156 ± 0.0005	0.8539 ± 0.0012	0.7583 ± 0.0019	0.9112 ± 0.0006	0.8241 ± 0.0016	0.6981 ± 0.0031	0.9150 ± 0.0008	0.8132 ± 0.0016	0.6852 ± 0.0022
Donoho [18]	0.9123 ± 0.0005	0.8527 ± 0.0012	0.7586 ± 0.0019	0.9041 ± 0.0006	0.8210 ± 0.0016	0.6976 ± 0.0032	0.8998 ± 0.0009	0.8043 ± 0.0015	0.6837 ± 0.0021
Immeakar [10]	0.9129 ± 0.0004	0.8528 ± 0.0012	0.7589 ± 0.0019	0.9064 ± 0.0005	0.8219 ± 0.0015	0.6975 ± 0.0032	0.9012 ± 0.0008	0.8064 ± 0.0014	0.6837 ± 0.0021
S. I. Olsen [8]	0.9158 ± 0.0005	0.8548 ± 0.0012	0.7511 ± 0.0025	0.9115 ± 0.0006	0.8264 ± 0.0016	0.6984 ± 0.0031	0.9122 ± 0.0011	0.8135 ± 0.0018	0.6879 ± 0.0021
S. Pyatykh [28]	0.9152 ± 0.0005	**0.8540**± 0.0012	0.7517 ± 0.0020	0.9107 ± 0.0006	0.8246 ± 0.0016	0.6985 ± 0.0031	0.9122 ± 0.0009	0.8139 ± 0.0015	**0.6859** ± **0.0021**
Our proposed	**0.9159** ± **0.0005**	**0.8540** ± **0.0009**	**0.7580** ± **0.0018**	**0.9128** ± **0.0006**	**0.8251** ± **0.0014**	**0.6983** ± **0.0032**	**0.9197** ± **0.0005**	**0.8172** ± **0.0013**	0.6856 ± 0.0017
**BM3D [43] +**		***Cactus***			***Desert***			***Koala***	
**Predictive Model**	**10**	**20**	**40**	**10**	**20**	**40**	**10**	**20**	**40**
True noise level (benchmark)	0.9049 ± 0.0005	0.8008 ± 0.0014	0.6805 ± 0.0019	0.8786 ± 0.0007	0.8029 ± 0.0010	0.7222 ± 0.0015	0.9117 ± 0.0005	0.8166 ± 0.0013	0.6957 ± 0.0027
Tai Yang [14]	0.9010 ± 0.0008	0.7975 ± 0.0019	0.6799±0.0020	0.8750 ± 0.0012	0.8020 ± 0.0011	0.7226 ± 0.0016	0.9110 ± 0.0006	0.8158 ± 0.0013	**0.6956**± 0.0027
Donoho [18]	0.8853 ± 0.0008	0.7905 ± 0.0014	0.6789 ± 0.0020	0.8687 ± 0.0009	0.8006 ± 0.0011	0.7228 ± 0.0015	0.9074 ± 0.0007	0.8141 ± 0.0013	0.6955 ± 0.0027
Immeakar [10]	0.8895 ± 0.0007	0.7927 ± 0.0015	0.6788 ± 0.0020	0.8696 ± 0.0008	0.8005 ± 0.0010	0.7233 ± 0.0014	0.9091 ± 0.0006	0.8144 ± 0.0013	0.6951 ± 0.0027
S. I. Olsen [8]	0.8994 ± 0.0007	**0.8011** ± **0.0015**	0.6819 ± 0.0020	0.8766 ± 0.0010	0.8046 ± 0.0009	0.7141 ± 0.0019	0.9109 ± 0.0007	0.8184 ± 0.0013	0.6948 ± 0.0026
S. Pyatykh [28]	0.8997 ± 0.0009	0.7985 ± 0.0019	**0.6805** ± **0.0020**	0.8750 ± 0.0011	**0.8025** ± **0.0011**	0.7215 ± 0.0016	0.9111 ± 0.0006	**0.8167** ± **0.0013**	**0.6958**± 0.0027
Our proposed	**0.9056** ± **0.0006**	0.8000 ± 0.0014	0.6800 ± 0.0021	**0.8776** ± **0.0009**	0.8022 ± 0.0011	**0.7219** ± **0.0017**	**0.9115** ± **0.0005**	0.8150 ± 0.0017	**0.6958** ± **0.0024**

## Data Availability

The data presented in this study are available on request from the corresponding author. Data are not publicly available due to privacy considerations.
